# Monitoring of liver function in a 73-year old patient undergoing ‘Associating Liver Partition and Portal vein ligation for Staged hepatectomy’: case report applying the novel liver maximum function capacity test

**DOI:** 10.1186/s13037-016-0104-y

**Published:** 2016-06-10

**Authors:** Felix Oldhafer, Kristina I. Ringe, Kai Timrott, Moritz Kleine, Wolf Ramackers, Sebastian Cammann, Mark D. Jäger, Juergen Klempnauer, Hueseyin Bektas, Florian W. R. Vondran

**Affiliations:** Regenerative Medicine & Experimental Surgery (ReMediES); Department of General, Visceral and Transplant Surgery, Hannover Medical School, Carl-Neuberg-Str. 1, 30625 Hannover, Germany; Department of Diagnostic and Interventional Radiology, Hannover Medical School, Hannover, Germany

**Keywords:** ALPPS, In situ split, Liver, Surgery, Liver resection, LiMAx

## Abstract

**Background:**

The two-stage liver resection combining in situ liver transection with portal vein ligation, also referred to as ALPPS (Associating Liver Partition and Portal vein ligation for Staged hepatectomy), has been described as a promising method to increase the resectability of liver tumors. However, one of the most important issues regarding the safety of this procedure is the optimal timing of the second stage at the point of sufficient hypertrophy of the future liver remnant. The recently developed liver maximum function capacity test (LiMAx) can be applied to monitor the liver function postoperatively and hence could be a useful tool for decision-making regarding the timing of the second stage of ALPPS.

**Case presentation:**

A 73-year-old female patient presented with metachronous colorectal liver metastasis comprising the complete right liver lobe as well as segment IV. Due to an insufficient future liver remnant (19.3 %; segments II and III of the liver) and a low future liver remnant:body weight ratio (0.28 %) the decision was made to perform an ALPPS-procedure in order to avoid development of postoperative small-for-size syndrome. Despite a formally sufficient increase of the FLR to 30.8 % within 7 days after the first step of ALPPS, the liver function was seen to only slowly increase as expressed by a LiMAx value of 245 μg/h/kg (baseline of 282 μg/h/kg prior to surgery). By means of the LiMAx test, sufficient increase of liver function eventually was detected by postoperative day 11 (LiMAx value of 371 μg/h/kg; FLR 35.2 %) so that the second step of ALPPS (completion of hepatectomy) was performed with no signs of liver failure during further clinical course.

**Conclusion:**

Performing ALPPS we have observed a significant difference between the increase in future liver remnant volume and function applying the LiMAx test. The latter tool thus might proof valuable for application in two-stage liver resection to avoid postoperative small-for-size syndrome.

## Background

The recently developed strategy to perform a two-stage liver resection combining in situ liver transection with portal vein ligation, also known as the ALPPS-procedure (Associating Liver Partition and Portal vein ligation for Staged hepatectomy), has been described as a promising method to increase the resectability of marginally resectable or locally unresectable liver tumors [1]. Since the introduction of ALPPS, several groups worldwide have adopted this new technique mainly to enlarge the pool of patients with resectable colorectal liver metastases [2]. However, since the introduction of ALPPS the safety of this procedure regarding morbidity and mortality is discussed controversially [3]. One of the advantages of the ALPPS-procedure is the rapid and sufficient hypertrophy of the future liver remnant (FLR) induced by the first step of the operation [4, 5], therefore the right timing of the second step is vital for the success of this approach. The reference standard in the assessment of the FLR is a combination of CT-volumetry and blood tests including Quick value, cholinesterase (CHE), international normalized ratio (INR) and Bilirubin [6]. However, in the majority of cases the final quality of the FLR in terms of function remains pretty uncertain.

The likewise recently developed liver maximum function capacity test (LiMAx) [7] is based on the hepatocyte-specific metabolism of the ^13^C-labelled substrate methacetin by the cytochrome P450 1A2 enzyme, which is ubiquitously active throughout the liver. After i.v. injection, the ^13^C-methacetin is instantly metabolized into acetaminophen and ^13^CO_2_, which is pulmonarily exhaled. Hence, the administration of ^13^C-methacetin leads to a significant alteration of the normal ^13^CO_2_:^12^CO_2_ ratio (Pee Dee Belemnite standard 1.1237 %) [8] in the expired breath. This alteration is determined by a suitable device called *Fast Liver Investigation Package* (FLIP) which is connected to the patient. Based on the ^13^CO_2_:^12^CO_2_ ratio and the body weight of the patient, the cytochrome P450 1A2 activity is determined and expressed as the so-called LiMAx value with the units μg/kg/h (μg methacetin/kg body weight/hour). The analysis of the ^13^CO_2_:^12^CO_2_ ratio is performed over a period of 20 to 60 min. For normal liver function, a LiMAx value more than 315 μg/kg/h is required. In healthy volunteers, the normal range was found to be 425 ± 67 μg/kg/h (range: 311–575 μg/kg/h) [9, 10].

Performing ALPPS, the precise assessment of the FLR function seems to be more important than its volume alone to determine the optimal time of hepatectomy to avoid postoperative small-for-size syndrome [11]. The described liver function determined by the LiMAx test thus has the potential to help to define the right timing of the second step of the ALPPS-procedure. Here we report our experience with the LiMAx test in a patient who recently underwent the ALPPS-procedure.

## Case presentation

A 73-year old woman presented in our outpatient clinic with metachronous colorectal liver metastasis. Previously a right hemicolectomy had been performed for a pT3, N0, M0 tumor. The CT-scan showed a large, colorectal metastasis-typical lesion comprising the complete right liver lobe as well as segment IV. Preoperative staging CT-scan showed no extrahepatic tumor manifestations. The patient expressed a strong wish for surgical therapy and refused any neoadjuvant chemotherapy. Consequently, the indication for liver resection was set and planned as an extended right hepatectomy (resection of segments IV-VIII). However, conventional liver resection was considered as too hazardous as the FLR was found to be only 19.3 % of the total liver volume (325 cm^3^ and 1687 cm^3^, respectively), which was below the volume cut-off value for safe resections (>20 % of total liver volume) [12]. The FLR:body weight (BW) ratio (FLR/BW) was only 0.28 %. Furthermore, liver maximum function capacity determined by the LiMAx test was only 282 μg/h/kg, which is reported to be associated with a significantly increased morbidity and mortality after major liver resection (>315 μg/h/kg required) [7]. In contrast, conventional laboratory values showed to be sufficient for a major liver resection including Quick value (88 %), CHE (6.59 kU/l) and Bilirubin (5 μmol/l) [13, 14]. In conclusion, due to the low FLR volume, the low FLR/BW ratio and the low LiMAx value, it was decided to apply the ALPPS-procedure in order to induce hypertrophy of segments II and III prior to hepatectomy. ALPPS was preferred to conventional portal vein occlusion due to the known more rapid hypertrophy of the FLR and thus the expectation to lower the risk of tumor progression in this patient who refused preoperative chemotherapy [4, 5].

The operation was started by careful exploration of the abdominal cavity to rule out any extrahepatic manifestations followed by examination of the liver by palpation and ultrasound to confirm the known lesion and to finally evaluate operability. Then ALPPS was performed as previously reported [15]. In brief, the hepatoduodenal ligament was dissected to isolate the right hepatic artery, portal vein and bile duct. Then the right hepatic vein was isolated. After transection of the right portal vein, the hepatic parenchyma was dissected between segments II/III and segment IV. Small hepatic veins draining from the right liver into the vena cava likewise were transected. The right liver lobe was then wrapped in a silicone matting to prevent adhesions while waiting for hypertrophy of the FLR. The patient required 3 transfusions of packed red blood cells (pRBC) intraoperatively and received 2 packages of fresh frozen plasma (FFP) within the first 12 h postoperatively. Overall, she showed an uneventful postoperative recovery after the first step of the ALPPS-procedure. Nevertheless, due to the advanced age of the patient, development of postoperative oedema with mild pulmonary restriction, high levels of liver enzymes (see below) in conjunction with lack of experience with this type of surgical approach, the patient was monitored at the intensive care unit (ICU) for 7 days.

**Fig. 1:**
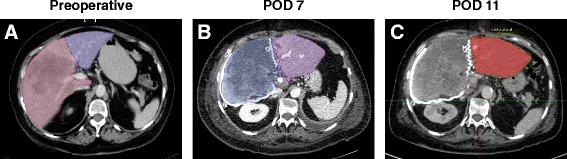
Pre- and postoperative CT-scan of the liver. **a** Preoperative CT-scan depicting the tumor lesion within the right liver lobe (marked pink) and the resulting future liver remnant (FLR; marked purple). **b** CT-volumetry 7 days after the first step of ALPPS already resulted in a significant increase of the FLR. The extended right liver lobe (wrapped in a silicone matting) meanwhile showed signs of necrosis following ligation of the right portal vein. **c** CT-volumetry 11 days after the first step of ALPPS showed further growth of the FLR (marked red). POD = postoperative day

**Fig. 2:**
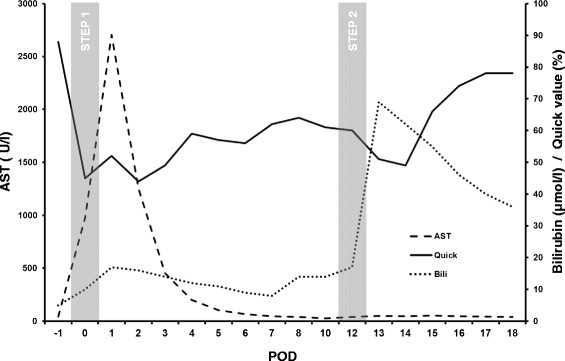
Postoperative course of AST, Bilirubin and Quick value. Diagram depicting the courses of AST, Bilirubin and Quick value following the first and second step of the ALPPS-procedure, respectively. POD = postoperative day

A CT-based volumetry on postoperative day (POD) 7 showed an increase of FLR volume to 519 cm^3^ representing approx. 30.8 % of the total liver volume with a FLR/BW ratio of 0.45 (Fig. 1). However, the LiMAx test revealed a functional capacity of still only 245 μg/h/kg. Therefore the second step of the ALPPS-procedure was delayed waiting for further hypertrophy and functional uptake of the FLR. At this time the conventional liver function values such as Quick value and Bilirubin already recovered well but still did not reach their baseline. AST and ALT levels were primarily significantly elevated to 2705 U/I and 2187 U/I on the first POD, respectively, but constantly dropped to 46 U/I and 229 U/I on POD 7. The courses of these laboratory values are depicted in Fig. 2.

**Fig. 3:**
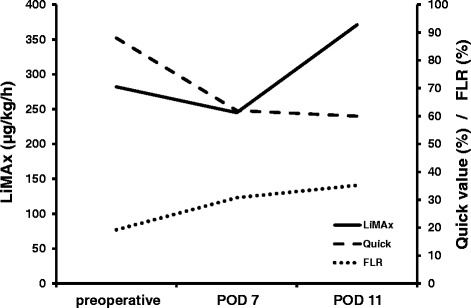
Postoperative course of FLR, LiMAx value and Quick value. Diagram depicting the courses of FLR, LiMAx value and Quick value following the first step of the ALPPS-procedure, respectively. POD = postoperative day

A further CT-volumetry conducted on POD 11 showed a FLR volume of 594 cm^3^ (approx. 35.2 % of the total liver volume; FLR/BW ratio of 0.52) resulting in a volume increase of about 82.8 % following step 1 of ALPPS. At this time also a recovery of liver function as monitored amongst others by the LiMAx test was observed. The latter now was found to be 371 μg/h/kg indicating sufficient growth and function of the FLR (Fig. 3). Thus, the second step of ALPPS was realized on POD 12. The mobilization of the liver was performed without any difficulties. The silicon matting covering the liver was removed; right hepatic artery, right hepatic bile duct and right hepatic vein were dissected. There was no major intraoperative complication and no units of pRBC or FFP had to be transfused and the patient could be discharged from ICU on POD 2.

Following the second step of ALPPS, the Quick value again significantly dropped and Bilirubin rised on POD 1, but recovered rapidly thereafter. Notably, on POD 3 the bilirubin was higher than 50 μmol/l and the Quick value below 50 % indicating a high risk for post hepatectomy liver failure [6]. Nonetheless, after completion of ALPPS the patient showed a stable liver function throughout the entire postoperative course. AST and ALT levels were only slightly elevated (Fig. 2). Unfortunately though, the patient developed a peripheral bile leak. Applying endoscopic sphincterotomy and biliary stenting the bile leak was successfully treated and dissolved within a few days. The abdominal drainages thus could be removed close by. Eventually, the patient continually recovered from these procedures and could be discharged on POD 30 after completion of ALPPS.

Due to the above mentioned oncological diagnosis an adjuvant chemotherapy with FOLFOX was recommended by the interdisciplinary tumor board. The last oncological staging was performed 12 month after ALPPS with no signs of tumor recurrence.

## Discussion

Mortality after the second step of the ALPPS-procedure mainly is related to liver failure, therefore insufficient FLR is one of the major challenges of ALPPS. The widely accepted cut-off values to perform the second step are FLR > 30 % (FLR/BW > 0.5 %) or > 40 % (FLR/BW > 0.8 %) depending on the parenchymal quality of the liver [12]. The lately published recommendations from the first International ALPPS Expert Meeting in Hamburg, Germany, states that the first CT-scan should be performed 8 days after step one of the procedure [11] since the FLR growth reaches a plateau after day 7 [16]. Furthermore, if there are any signs of liver failure, step 2 should be delayed even when sufficient FLR volume has been reached. The reliability of function tests such as the Indocyanine Green (ICG) test, the (99 m) Tc-mebrofenin hepatobiliary scintigraphy (HBS) with SPECT-CT or the LiMAx test have not been studied sufficiently, yet, in the setting of ALPPS. Therefore, the degree of hypertrophy achieved after step 1 remains the main criteria for decision-making about the timing of the second step of the procedure, even though the correlation between volumetric and functional increase of the FLR after ALPPS is still a matter of debate.

We here demonstrate our preliminary experience with the LiMAx test in combination with the conventional CT-volumetry of the liver to assess liver regeneration in the case of an ALPPS-procedure. We were able to show a substantial difference between the increases in FLR volume and function applying the LiMAx test (Fig. 3). When we performed the first evaluation of FLR function and growth 7 days after the first stage of the resection, the FLR volume had increased from 19.3 to 30.8 % indicating sufficient growth to undergo the next step of the operation as the cut-off value for safe resection was reached [12]. However, the FLR function quantified by the LiMAx test was only 245 μg/h/kg, which is reported to be associated with high morbidity and mortality after major resection [7]. Only four days later, the FLR function had sufficiently increased to 371 μg/h/kg and the next step of the resection could be performed safely. Therefore, we can report the same experience as Cieslak et al. using the (99 m) Tc-mebrofenin HBS with SPECT-CT to measure liver function early after the first stage of ALPPS that the volume of the FLR might overestimate its function [17]. Hence, there is a considerable risk to induce postoperative small-for-size syndrome when decision-making regarding the second stage of the ALPPS-procedure is based on volume increase only.

Lately Malinowski et al. published their experience with the LiMAx test for liver resection following portal vein embolization (PVE) which at least partly has the same physiological background to induce liver hypertrophy as the ALPPS-procedure. They showed that the LiMAx value did not change rapidly after PVE, and between PVE and hepatectomy there was also only an insignificant increase in the LiMAx value (on average from 360 to 401 μg/kg/h) showing a slow and homogeneous remodeling process after the intervention [18]. Thus, in conjunction with our present case, this again might demonstrate the advantage of ALPPS over PVE regarding a more rapid growth in liver volume and function, and is in line with the conclusions drawn by others [19].

However, since the LiMAx test represents a global liver function test just like the ICG test, there are also some limitations. In contrast to imaging-based liver function testing, global liver function tests have the disadvantage that regional dysfunction or heterogeneous distribution of liver function cannot be displayed. Especially in the case of ALPPS it is assumed that liver function after the first step of the procedure is no longer distributed equally over the whole liver and therefore the prediction of postoperative liver function remains challenging. The imaging based liver function tests such as the (99 m) Tc-mebrofenin HBS has the potential advantage that liver function can be displayed separately for the right and left lobe or as in the case of ALPPS for the FLR as recently shown [17]. On the other hand, current imaging based liver function tests are not available for routine clinical use and are much more cost and time intensive as compared to the LiMAx test which is comparable easy to use and could be installed ubiquitously much more readily.

## Conclusion

**Fig. 4:**
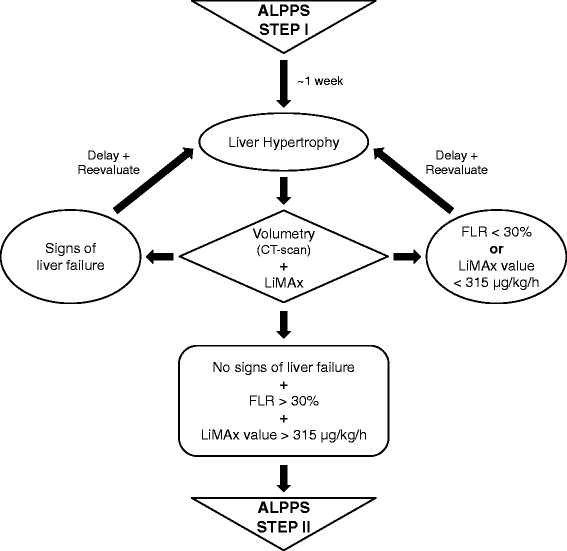
Algorithm summarizing the recommendations for performance of ALPPS. Diagram summarizing the suggestions of the first International ALPPS Expert Meeting in Hamburg [11] modified by possible implementation of the LiMAx test

LiMAx is a simple liver function test enabling monitoring of FLR function in patients undergoing the ALPPS-procedure. LiMAx thus has the potential to be a useful tool in defining the optimal timing of the second stage of ALPPS (Fig. 4) and hence might contribute substantially to the improvement of the procedure-related morbidity and mortality rates.

## Abbreviations

ALPPS, Associating Liver Partition and Portal vein ligation for Staged hepatectomy; CHE, cholinesterase; FFP, fresh frozen plasma; FLIP, Fast Liver Investigation Package; FLR, future liver remnant; FLR/BW ratio, future liver remnant/body weight ratio; HBS, hepatobiliary scintigraphy; ICG, Indocyanine Green; ICU, intensive care unit; INR, international normalized ratio; LiMAx, liver maximum function capacity test; POD, postoperative day; pRBC, packed red blood cells; PVE, portal vein embolization.
